# Clustering of four major lifestyle risk factors among Korean adults with metabolic syndrome

**DOI:** 10.1371/journal.pone.0174567

**Published:** 2017-03-28

**Authors:** Shin Ha, Hui Ran Choi, Yo Han Lee

**Affiliations:** 1 Department of Public Health, Graduate school of Korea University, Seoul, South Korea; 2 Services Department, Medical Library, Korea University, Seoul, South Korea; 3 Division of Family Medicine, Seoul National University Graduate School of Medicine, Seoul, South Korea; 4 Department of Preventive Medicine, Konyang University College of Medicine, Daejeon, South Korea; The University of Tokyo, JAPAN

## Abstract

The purpose of this study was to investigate the clustering pattern of four major lifestyle risk factors—smoking, heavy drinking, poor diet, and physical inactivity—among people with metabolic syndrome in South Korea. There were 2,469 adults with metabolic syndrome aged 30 years or older available with the 5th Korean National Health and Nutrition Examination Survey dataset. We calculated the ratio of the observed to expected (O/E) prevalence for the 16 different combinations and the prevalence odds ratios (POR) of four lifestyle risk factors. The four lifestyle risk factors tended to cluster in specific multiple combinations. Smoking and heavy drinking was clustered (POR: 1.86 for male, 4.46 for female), heavy drinking and poor diet were clustered (POR: 1.38 for male, 1.74 for female), and smoking and physical inactivity were also clustered (POR: 1.48 for male). Those who were male, younger, low-educated and living alone were much more likely to have a higher number of lifestyle risk factors. Some helpful implications can be drawn from the knowledge on clustering pattern of lifestyle risk factors for more effective intervention program targeting metabolic syndrome.

## Introduction

Metabolic syndrome is a clustering of cardio-metabolic risk factors. People with metabolic syndrome are much more likely to have cardiovascular disease (CVD) and type 2 diabetes, respectively than people without metabolic syndrome [1–3]. The prevalence of metabolic syndrome has steadily increased and is now a serious public health concern in developed countries [4,5].

There is definite epidemiological evidence that major four lifestyle risk factors such as smoking, heavy drinking, poor diet, and physical inactivity contribute to the development of metabolic syndrome and chronic diseases [6,7]. Researches also suggest that these lifestyle risk factors are not randomly distributed, but that they tend to cluster with other unhealthy behaviors within individuals [8,9]. In other words, certain combinations of lifestyle risk factors is more prevalent than can be expected based on the prevalence of individual lifestyle risk factors.

Although there have been several studies on the clustering of multiple lifestyle risk factors in the general population [8–10], very little efforts for obtaining relevant information on people with metabolic syndrome had been done. Knowledge on unhealthy behavior pattern of people with metabolic syndrome is considered crucial for creating a tailored lifestyle intervention program and a tailored regimen might help patients with metabolic syndrome to be motivated to change unhealthy behaviors [11,12]. The purpose of this study was to investigate the clustering pattern of four major lifestyle risk factors—smoking, heavy drinking, poor diet, and physical inactivity—among people with metabolic syndrome in South Korea.

## Methods

### Study population

Data for this study were drawn from the population-based, nationwide cross-sectional study of the 5th Korean National Health and Nutrition Examination Survey (KNHANES) 2010–2012, which was conducted by the Korea Centers for Disease Control and Prevention (KCDC). A multistage, stratified, probability sampling approach was used with selections made from sampling units based on gender, age group and geographical area using household registries. The methods have already been detailed in previous articles [13,14].

There were 2,918 adults with metabolic syndrome aged 30 years or older available with the 5th KNHANES dataset. We excluded 449 subjects who had missing data for any of four lifestyle risk factors. Finally, 2,469 participants were included in the analyses. The American Heart Association/National Heart, Lung, and Blood Institute Scientific Statement defined the criteria for metabolic syndrome in Asians as follows and the criteria was applied for this study [1]. Subjects with 3 or more of the following 5 metabolic derangements were defined as having metabolic syndrome: abdominal obesity more than 90 cm in men and more than 80 cm in women, blood pressure more than 130/85 mm Hg or the use of an antihypertensive drug, fasting glucose more than 100 mg/dL or the use of an antidiabetic drug, an high-density lipoprotein cholesterol level less than 40 mg/dL in men and 50 mg/dL in women or the use of an antidyslipidemic drug, and a high-density lipoprotein cholesterol level more than 150 mg/dL or use of an antidyslipidemic drug.

Written informed consent was provided by all participants, and the protocol for the survey was approved by the Institutional Review Board of the KCDC (2010-02CON-21-C, 2011-02CON-06-C, 2012-01EXP-01-2C). The current study did not require additional ethical approval because the KNHANES dataset is publicly available.

### Measurements

Data on smoking status, alcohol intake, dietary intake and physical activity were self-reported. Smoking status was divided into two categories: current smoker and ex- or never-smoker. Heavy drinking was defined as alcohol intake ≥ 5 glasses for male and ≥ 4 glasses for female subjects on one occasion ≥ once a week. The definition of physical inactivity was adapted from the International Physical Activity Questionnaire (IPAQ) category 1 in which no activity is reported or some activity is reported but not enough to meet an equivalent of 30 min of at least moderate intensity physical activity on >3 days per week [15,16]. Dietary intake was assessed by the 24-h dietary recall method. Poor dietary patterns were defined as having one or two of the following two components: fat intake exceeding 30% of the total number of calories, and sodium intake exceeding 30% of the average daily intake of Koreans. Socioeconomic factors such as income, education level, marital status and job state were used for analysis.

### Statistical analyses

The analyses in this study consisted of three parts. First, we calculated the ratio of the observed to expected (O/E) prevalence for the 16 different combinations of four lifestyle risk factors. O/E ratio presents both the magnitude and direction of the association, with values above 1 meaning positive association and below 1 an inverse association [17]. The more the ratio deviates from 1, the stronger the behaviors are associated. In other words, clustering exists when the observed prevalence of a certain combination of lifestyle risk factors exceeds the expected prevalence of the combination.

Second, we examined the associations between sets of two lifestyle risk factors by calculating the prevalence odds ratios (POR) and statistically tested by Chi-square tests. If the 95% confidence interval of the POR does not include 1, it indicates clustering between the two lifestyle risk factors. The POR was calculated as follows: POR = (number of respondents without with both risk factors) ÷ (number of respondents with the one risk factor × number of respondents with the other risk factor). Analyses were performed separately for male and female subjects for the former two parts of analyses because the prevalence of smoking, heavy drinking, and poor diet were substantially different for male and female subjects.

Third, we examined the sociodemographic patterns in the presence of multiple lifestyle risk factors. Multinomial logistic regression model was used to evaluate the association between sociodemographic variables and the dependent variable, the number of lifestyle risk factors, ranging from 0 to 4. The model allows us to estimate the probability that a respondent has a certain number of lifestyle risk factors (1, 2, 3, and 4) compared to the reference groups of having no risk factors. All analyses were carried out using Stata 11.1 (StataCorp, College Station, TX, USA).

## Results

Table 1 shows the characteristics of the study population. Compared with male subjects, females tended to be older, in the lower income, less educated, living alone, and not employed. Males were highly likely to be smokers, heavy drinkers, and to have poor diet than female subjects, whereas the prevalence of physical inactivity was similar in both sexes. Of the males, 11% had no lifestyle risk factor, 26% had one, 37% had two, and 26% had simultaneously three or more lifestyle risk factors. Meanwhile, more than 80% of female participants had nothing or only one lifestyle risk factor.

**Table 1:** Characteristics of the study population, n (%).

	Men (n = 1,197)	Women (n = 1,272)	p-value
Age group			<0.001
30s	179 (15.0)	91 (7.2)	
40s	278 (23.2)	205 (16.1)	
50s	360 (30.1)	414 (32.6)	
60s	380 (31.8)	562 (44.2)	
Smoking	504 (42.1)	51 (4.0)	<0.001
Heavy drinking	512 (42.8)	100 (7.9)	<0.001
Poor diet	551 (46.0)	272 (21.4)	<0.001
Physical inactivity	641 (53.6)	724 (56.9)	0.100
Number of lifestyle risk factors			<0.001
4	77 (6.4)	3 (0.2)	
3	238 (19.9)	24 (1.9)	
2	438 (36.6)	202 (15.9)	
1	310 (25.9)	659 (51.8)	
0	134 (11.2)	384 (30.2)	
Household income in quartiles			<0.001
Q1 (Lowest)	270 (22.9)	385 (30.5)	
Q2	320 (27.1)	335 (26.6)	
Q3	292 (24.8)	311 (24.6)	
Q4 (Highest)	298 (25.3)	231 (18.3)	
Education			<0.001
Primary school	188 (15.7)	599 (47.2)	
Middle school	192 (16.1)	219 (17.2)	
High School	404 (33.8)	326 (25.7)	
College or more	411 (34.4)	125 (9.8)	
Married status			<0.001
Living alone	121 (10.1)	266 (20.9)	
Living with partner	1,074 (89.9)	1,004 (79.1)	
Economic status			<0.001
Active	978 (82.4)	620 (48.9)	
Inactive	209 (17.6)	649 (51.1)	

Table 2 shows the observed and expected prevalence of all 16 possible combinations of the four lifestyle risk factors. In males, clustering was found at both ends of the lifestyle spectrum. In other words, the observed prevalence of having no, and having all four lifestyle risk factors was higher than could have been expected (O/E ratio 1.45 and 1.35, respectively) on the basis of the individual probabilities of the four risk factors alone. The observed prevalence of specific combinations such as smoking + heavy drinking + poor diet or smoking + heavy drinking + physical inactivity was higher than could have been expected. Since about 80% of the female participants had one or no risk factors, the prevalence of the majority of combinations were very low, and thus this made it difficult to interpret the O/E ratios for female subjects.

**Table 2:** Prevalence of combinations of four lifestyle risk factors.

No.	S	A	D	P	Men, n = 1,197			Women, n = 1,272		
					N	O (%)	O/E	N	O (%)	O/E
**4**	**+**	**+**	**+**	**+**	77	6.43	1.45	3	0.24	6.24
**3**	**+**	**+**	**+**	**-**	56	4.68	1.22	2	0.16	5.49
	**+**	**+**	**-**	**+**	80	6.68	1.28	3	0.24	1.70
	**+**	**-**	**+**	**+**	51	4.26	0.72	3	0.24	0.54
	**-**	**+**	**+**	**+**	51	4.26	0.70	16	1.26	1.36
**2**	**+**	**+**	**-**	**-**	47	3.93	0.87	5	0.39	3.64
	**+**	**-**	**+**	**-**	50	4.18	0.81	6	0.47	1.38
	**+**	**-**	**-**	**+**	90	7.52	1.08	18	1.42	0.86
	**-**	**+**	**+**	**-**	75	6.27	1.19	10	0.79	1.13
	**-**	**+**	**-**	**+**	76	6.35	0.89	30	2.36	0.70
	**-**	**-**	**+**	**+**	100	8.35	1.02	133	10.46	0.97
**1**	**+**	**-**	**-**	**-**	53	4.43	0.73	11	0.86	0.69
	**-**	**+**	**-**	**-**	50	4.18	0.67	31	2.44	0.95
	**-**	**-**	**+**	**-**	91	7.60	1.08	99	7.78	0.95
	**-**	**-**	**-**	**+**	116	9.69	1.01	518	40.72	1.03
**0**	**-**	**-**	**-**	**-**	134	11.19	1.35	384	30.19	1.01

Note: S, smoking; A, heavy drinking; D, poor diet; P, physical inactivity; O, observed; E, expected.

Table 3 shows the POR of combinations of two lifestyle risk factors. The overall pattern of clustering was similar for males and females. Clustering of smoking with heavy drinking was observed (POR 1.86 for male and 4.46 for female subjects). In addition, heavy drinking and poor diet were clustered in both sexes. In other words, the participants who smoke are more likely have heavy drinking, and the participants who drink heavily are more likely have poor diet. In male participants, smoking and physical inactivity were also clustered showing POR with 1.48.

**Table 3:** Prevalence and Prevalence Odds Ratio (POR) of combinations of two lifestyle risk factors.

	Men			Women		
	P (%)	POR	P value	P (%)	POR	P value
Smoking/Heavy drinking	260 (21.7)	1.86	<0.001	13 (1.0)	4.46	<0.001
Smoking/Poor diet	234 (19.6)	1.03	0.814	14 (1.1)	1.41	0.283
Smoking/Physical inactivity	298 (24.9)	1.48	0.001	27 (2.1)	0.85	0.559
Heavy drinking/Poor diet	259 (21.6)	1.38	0.006	331 (2.4)	1.74	0.016
Heavy drinking/Physical inactivity	284 (23.7)	1.14	0.250	52 (4.1)	0.81	0.302
Poor diet/Physical inactivity	279 (23.3)	0.80	0.062	155 (12.2)	1.00	0.980

Note: POR, prevalence odds ratio.

Table 4 presents the results of the multinomial logistic regression model, with the number of lifestyle risk factors as the dependent variable. The table shows that male and younger age groups were much more likely to have a higher number of lifestyle risk factors. Those who were low educated or living alone were also more likely to have multiple lifestyle risk factors, while other important socioeconomic factors such as income and economic status were not significantly associated with the number of lifestyle risk factors.

**Table 4:** Odds ratios (OR) and 95% confidence intervals (CI) of predictors of the number of lifestyle risk factors.

	1		2		3		4	
	OR	95% CI	OR	95% CI	OR	95% CI	OR	95% CI
Sex (women)								
Men	1.40	1.07–1.84	6.54	4.84–8.86	27.11	16.24–45.224	73.77	21.79–249.78
Age group (60+)								
50s	1.08	0.83–1.40	1.51	1.11–2.07	2.27	1.42–3.631	6.42	2.45–16.83
40s	1.72	1.17–2.54	3.09	2.00–4.77	8.13	4.63–14.28	24.94	8.74–71.14
30s	1.36	0.81–2.28	4.31	2.52–7.40	9.07	4.64–17.73	17.75	5.47–57.56
Income (Q1)								
Q2	0.91	0.68–1.24	1.17	0.83–1.65	0.75	0.46–1.21	1.09	0.54–2.21
Q3	0.95	0.70–1.29	0.90	0.63–1.29	0.81	0.49–1.30	0.54	0.24–1.21
Q4	0.93	0.67–1.30	1.02	0.70–1.51	0.99	0.60–1.62	0.92	0.43–1.98
Education (primary)								
Middle school	0.90	0.65–1.24	1.05	0.72–1.54	0.89	0.49–1.60	0.58	0.22–1.55
High School	0.92	0.67–1.26	0.99	0.68–1.42	0.99	0.58–1.69	0.44	0.18–1.07
College or more	0.82	0.54–1.24	0.60	0.38–0.97	0.58	0.31–1.09	0.55	0.22–1.39
Married status (living with partner)								
Living alone	0.98	0.72–1.31	1.43	1.00–2.03	1.64	0.99–2.71	2.20	1.05–4.63
Economic status (active)								
Inactive	0.95	0.75–1.20	0.85	0.64–1.13	0.63	0.39–1.00	0.69	0.30–1.59

Note: Reference category for the outcome variable is “none” lifestyle risk factor. Reference group is in parenthesis.

## Discussion

In the present study we investigated the clustering of these four major lifestyle risk factors such as smoking, heavy drinking, poor diet, and physical inactivity among a representative sample of adult Korean population with metabolic syndrome. Most previous studies on clustering of risk factors have examined the clustering of metabolic risk factors [18–25] and not lifestyle risk factors.

This study shows that the four lifestyle risk factors tended to cluster in specific multiple combinations. Smoking and heavy drinking was clustered, heavy drinking and poor diet were clustered, and smoking and physical inactivity were also clustered. These clustering patterns were extensively reported earlier for general population [8,9,26]. But this study failed to show the clustering of heavy drinking and high physical inactivity in the metabolic syndrome patients, like earlier reports for general population which showed clustering of heavy drinking and high physical activity [8,9].

It appeared that multiple lifestyle risk factors were more prevalent among men, low educated people and singles. High education may have positive effect on reducing number of having lifestyle risk factors [27,28]. Subjects educated above high school have lower possibility of having three or four risk factors than subjects with primary of middle school education. Female participants tended to be passive in terms of health behaviors in that they do not engage in smoking, heavy drinking, poor diet but as well as physical activity. About 40% of females were the case. This finding is consistent with the previous results that general Korean women tended not to partake in smoking, drinking, and physical activity at the same time [26]. Likewise, male participants tended to smoke and drink heavily, also supporting existing literature [26]. Males with metabolic syndrome were much more likely than their female counterparts to engage in multiple unhealthy behaviors. This results show that there are vulnerable groups within the adult population that have more risky lifestyles.

The crucial management approach for metabolic syndrome is not drug treatment, but lifestyle change, that is to say, making healthier lifestyle behaviors. The National Cholesterol Education Panel Adult Treatment Panel recommended lifestyle change as the initial management for metabolic syndrome [1,29]. Studies have shown that short-term intensive lifestyle intervention programs are effective at controlling risk factors for metabolic syndrome. For example, diet and exercise programs significantly decrease biochemical indices of metabolic syndrome, weight, and waist circumference [30–32]. A successful lifestyle intervention for the metabolic syndrome depends much on the patient's motivation to change their unhealthy behaviors [33]. Naturally, the more closely a patient adheres an intervention program, the greater the improvement in level of his or her lifestyle risk factors [34].

Followings are some helpful implications for more effective lifestyle intervention targeting metabolic syndrome from this study. First, multiple-behavior interventions should be provided rather than single-behavior interventions [35,36] because health behaviors are clustered. This approach can be more effective and efficient at achieving intervention goal [37]. Second, interventions need to focus on modifying smoking and heavy drinking habits. People who smoke and/or drink heavily tend to have other bad lifestyle habits. In other words, controlling for these two factors may prevent other bad habits. Third, men, younger age groups, low educated and singles should be targeted for multiple-behavior interventions because these groups appear to be relatively at high risk. Fourth, more tailored intervention should be developed for women with metabolic syndrome since most of female participants in this study have relatively good health habits. Further study is needed to identify behavior pattern among women with metabolic syndrome more precisely.

The most important limitation in the study is that as cut-off points of lifestyle risk factors were used that are in accordance with KNHANES, it may be difficult to generalize our findings to different settings and populations. It should be noticed that the common practice of dichotomizing lifestyle factors may have implications for the findings [8,38].

Generally, prevalence ratio (PR) is regarded more appropriate measure than POR, but the reason we used POR instead of PR was that only POR has the property of reciprocity (changing reference category of a dichotomized variable will yield ‘reciprocal’ estimates) which is needed for our analyses [39]. In fact, previous researches with similar study design used POR, too [8–10,17].

In the present study we examined the clustering of four major lifestyle risk factors among a representative sample of adult Korean population with metabolic syndrome. This study shows that the four lifestyle risk factors clustered in specific multiple combinations and there are specific vulnerable groups that have more lifestyle risk factors. Some helpful implications can be drawn from the knowledge of clustering pattern of lifestyle risk factors for more effective intervention program targeting metabolic syndrome.
